# Perception of Health Care Workers (HCWs) towards early antenatal booking in Fiji: A qualitative study

**DOI:** 10.1371/journal.pone.0276805

**Published:** 2022-11-28

**Authors:** Renita Maharaj, Masoud Mohammadnezhad

**Affiliations:** 1 Ba Mission Hospital, Ba, Fiji Islands; 2 School of Nursing and Healthcare Leadership, University of Bradford, Bradford, West Yorkshire, United Kingdom; World Health Organisation: Organisation mondiale de la Sante, INDIA

## Abstract

**Background:**

Early booking or registration into Antenatal Care (ANC) can be defined as initiation of ANC before 12 weeks of gestation and is important for the best health outcome of the mother and the baby. Delayed initiation of ANC has been linked to increased rate of maternal and fetal mortality. There is international consensus that ANC should begin within first trimester yet pregnant women delay initiation of ANC. Health Care Workers (HCWs) understanding of reasons for this can improve patient provider relationship.

**Objectives:**

This study aims to explore the perception of the HCWs in Fiji towards early antenatal booking.

**Methods:**

A qualitative study was employed using four Focus-Group Discussions (FGDs)with the HCWs who provide health care service for pregnant women in Ba Mission Hospital (BMH) in 2020. Each group comprised of medical officers, mid-wives and registered nurse who were chosen purposively. A semi-structured open ended questionnaire was used to guide the discussion. Data was transcribed and analyzed manually using thematic content analysis using the following process: familiarization, coding, identifying themes, reviewing and refining, integration and interpretation.

**Results:**

There was a total of 18 HCWs for the FGDs. The mean age of the participants was 37.4±11.8years. The three themes identified were: knowledge of HCWs on early booking, their perceived barriers and perceived enabling factors to early antenatal booking. The FGDs identified that the HCWs had adequate knowledge on early initiation of ANC and that there were a range of barriers to early initiation of ANC. The HCWs also suggested factors that could enable women to book early.

**Conclusion:**

Based on the study it can be concluded that the HCWs have a positive perception of early antenatal booking, however, there are various factors that contribute to delayed antenatal booking. The barriers to early ANC are both an opportunity and a challenge to strengthen and review the maternal services offered. The enabling factors should be reinforced from an individual level to the health system and the general context. The implications of the barriers and enabling factors identified in this study is to implement evidence-based policies to improve early antenatal booking in Ba, Fiji.

## Introduction

According to WHO and National Institute for Health and Clinical recommendations; all pregnant women should initiate ANC booking during the first trimester [1, 2]. A pregnant woman should visit the health facility as soon as she suspects that she is pregnant to receive antenatal care. Early initiation will facilitate screening for complications and tests that are most effective early in the pregnancy such as correct assessment of Estimated Date of Delivery (EDD) for correct treatment of preterm labor, provision of folic supplements to prevent neural tube defects, screening and treatment of iron deficiency anemia, Sexually Transmitted Diseases (STDs) and screening for congenital disorders [1–5]. Pregnant women are exposed to health education and counselling, the expected physiological changes in pregnancy and risk factors to adverse pregnancy outcomes during ANC visits. In addition, they get dietary counselling and immunization against tetanus and pre-existing medical condition such as hypertension and diabetes are detected during the booking visit [5, 6]. Early ANC booking might reduce maternal mortality and morbidity directly through detection and treatment of pregnancy related or intercurrent illness and indirectly through detection of women with increased risk of complications of delivery [4, 6–8].

All government health care facility offers free ANC service in Fiji. In Fiji it is recommended for women to seek ANC service as soon as they suspect pregnancy. All pregnant women are encouraged to attend at least four ANC visits in order to reduce the risk of adverse obstetric outcomes. Fiji is yet to adopt the World Health Organization (WHO) recommendations on ANC and currently the basic schedule of antenatal visit for a healthy mother is at booking (before 12weeks), 18, 28, 36, 37–38 and 41 weeks. At least four ANC visits are recommended. Late booker is defined as one who books after 24 weeks of gestation [5]. Ultrasound scans for morphology, growth scan and Amniotic Fluid Index for postdates women are done on subsequent visits. Moreover, screening test such as polycose test for Gestational Diabetes (GDM) and blood tests such as hemoglobin and VDRL are repeated later during pregnancy if indicated [5].

Despite the global progress that has been made in terms of access to ANC, high number of maternal death and stillbirths continue to occur [9]. About 2.6 million stillbirths occur annually and 830 maternal deaths occur daily globally due to pregnancy related problems [10], 1% of which occur in developed countries and 99% occur in developing countries [2, 4, 9, 11]. Estimates suggest that the regions with low rates of early ANC coverage are the ones with highest rate of Maternal Mortality Ratio (MMR). The life time risk of a woman’s death due to pregnancy or its complication is 1 in 180 in developing countries and 1 in 4900 in developed countries [12]. There is a strong positive association between the level of care during pregnancy and the use of safe delivery care which might explain how ANC reduces maternal mortality [2, 13, 14]. Maternal death and morbidity remain an on-going challenge for developing countries including Fiji.

The MMR for Fiji gradually fell from 49 deaths per 100,000 live births in 1996 to 30 deaths per 100,000 live births in 2015 [15]. However, an increase in MMR were reported as one of the major health issues in Fiji in 2014. One of the ways of tackle this issue is through enhancement of early initiation of ANC. Even though ANC coverage in Fiji has reached more than 95% with many mothers achieving more than four visits, less than 10% of women are booked in the first trimester [16]. When a woman initiates ANC late, opportunities to provide information and other interventions are missed [17]. Therefore, late booking is a catastrophic event in the health agenda. Despite the government’s effort to improve maternal and child health, the number of women who book late are increasing. Late booking does not only impact the mother and the baby but also impact the health system, the health care providers and the care they are able to give to pregnant mothers [18]. Therefore, it is important to understand the factors that contribute to late ANC booking in our setting.

Health Care Workers (HCWs) play a vital role in antenatal, delivery and postpartum care. Negative behaviors and attitudes of the HCWs are factors contributing to postponement of early ANC attendance [19]. However, little research has been done to explore HCWs understanding of patients’ care-seeking behavior. The perception of HCWs are important because their attitude and behavior influence health care seeking and quality of care both negatively and positively [20]. Patient provider relationship had a significant effect in the prevention of mother-to-child transmission of HIV in a study conducted in South Africa [21]. Setting-specific insights into ANC attendance related to cultural contexts and beliefs about pregnancy and childbirth can be provided by qualitative studies [22, 23]. To date, there has been no study conducted in Fiji to investigate the barriers to late ANC booking.

Due to paucity of information on HCWs understanding of the barriers and motivators of health seeking behaviors of women, this study aimed to explore the insight of HCWs into the women’s reasons for starting ANC late in Ba Mission Hospital, Fiji. The current study will be first of its kind in the Pacific and in Fiji that will address the knowledge gap and will build on existing literature by ascertaining findings from the current reviewed articles by looking at issues that are still unclear in relation to factors which delays early booking by pregnant mothers. Furthermore, government policy makers and stakeholders need to understand the factors that influence timely initiation of ANC for appropriate policy formulation and advocacy. There is a lack of evidence locally which leads to policy gaps. The current research can help in filling in some of these gaps.

## Methodology

### Study design and setting

A qualitative research was conducted among HCWs who provide ANC services in Ba Mission Hospital (BMH) from 12^th^ March to 2^nd^ April, 2020. BMH is the Sub-Divisional Hospital (SDH) of Ba where majority of the pregnant women seek ANC services. Ba is a district located in the Western side of Viti Levu, Fiji’s main island and has an agricultural center. It has an estimated population of around 55,000 with Fijians of Indian descent (68.5%) making up the largest proportion. The Child Bearing Age (CBA) population of Ba is about 19% of the total population (10,387) with Indo-Fijian (68.5%) comprising the majority of CBA population [23]. The health care service is provided through a three tiers system: nursing stations, health center and the SDH. The total number of mothers who were booked in BMH in 2020 was 692. Majority of the mothers booked were I-taukei (67.7%) followed by Fijians of Indian descends (30.2%) and only 2% of mothers booked were of other ethnicity (Rotuman, Chinese). Out of the total mothers who were booked in 2020 only about 26% of the mothers were booked in first trimester. The per cent of women booked in second trimester was 54% and third trimester booking was 30% [24, 25].

In Fiji, after confirmation of pregnancy, a pregnant woman will visit her nearest health facility, where her pregnancy will be registered by the area Medical officer or the nurse. She will have one ANC folder which will be kept by the health unit and one ANC attendance record which will be retained by her. The ANC folder will capture her socio-demographic data, clinical and past obstetric history as well the health of mother and the fetus on subsequent visits. Pregnant women are referred to BMH if any complication arises or if they are around term. Most of the mothers choose to seek ANC at BMH as it is convenient to them as the health center and nursing station does not have the basic investigation such as laboratory or ultrasound scan. Antenatal booking clinic in BMH is done on every Thursdays from 8am– 12pm and provision of service is for free. The booking is done by doctors, nurses and midwives. Apart from booking through clinic, women are also booked when they are seen in general outpatient with medical conditions or in prep room (where booked pregnant mothers routinely come with ailments).

### Study sample

All HCWs who provide ANC service in BMH formed the population of the Focus Group Discussions (FGDs). The total numbers of HCWs who provide services to pregnant mothers in BMH are 27 (15 medical officers, 14 mid-wives and 8 registered nurses). Homogenous purposeful sampling was used to recruit HCWs for the study with the aim of having representation of the various ANC health care providers with four groups of 5 participants in each. HCW of any gender, age or ethnicity who was either a doctor, nurse or a midwife; had worked with pregnant women (in one of the following: ANC clinic, ANC ward, prep room or delivery rooms) for at least one year were considered eligible for the study. Those who were not willing to participate and had less than one year of experience in ANC service provision were excluded from the study. The HCWs were included on the basis that that they have been working and interacting with the pregnant women and could provide information on their perception and views regarding early antenatal booking. There was a total of four FGDs with four to five members in each. The number of FGDs was based on the data saturation. Two groups comprised of five members (2 Mid-wives, 2 medical officers and 1 Medical officer) and another two comprised of four members only as 2 medical officers did not turn up for the FGDs (2 mid-wives, 1 medical officer and 1 nurse). Those interested were approached in person and were asked for their availability before an appointment was scheduled. The FGDs was conducted in a separate room away from the ANC clinic in the hospital setting and there was no compensation for the participants as the research was not sponsored.

The HCWs were taught in nursing and medical schools about ANC. For the past years there are divisional and in service trainings conducted on EMNOC. Ba Mission Hospital has an ANC guideline which is in line with the National Obstetric Guideline of Fiji.

### Data collection tool

A topic guide (Annex) was used for the FGDs to give detailed information and deep insight into the knowledge and perception of HCWs towards ANC and to further explore factors that affect timing of initiation of ANC. There were three sections that focused on: (i) demographic characteristics; (ii) 6 main/core questions that explored on knowledge, perception, barriers and facilitators of antenatal booking; (iii) probe and follow up questions were used for clarity and detailed exploration while ensuring credibility of the data. The questions were developed from several literature searches with key issues such as experience, knowledge and perception of antenatal booking.

### Study procedure

Prior awareness about the study was done to the ANC service providers and the time and place for the FGDs that was convenient to both the group and the researcher was decided in advance. They were reminded of the discussion two days before the scheduled meeting. The main researcher who was a female medical doctor with previous interviewing experience introduced herself as well as the topic of the research to establish credibility, the general purpose of the study, the aims of the interview and the expected duration, the format of the discussion and the importance of the cooperation of the participants was explained. A set of ground rules were set at the start of the discussion after which the information sheet was provided to the participants and consent was obtained. Demographic information of the participants was also obtained at this point.

The researcher was the moderator, taking a peripheral role in the discussion. The FGDs was conducted in English language s and there were no interpreters. The discussions were guided by the topic guide. Individual participants on the seating chair were allocated identification numbers for easier note taking and to key verbatim statements for keeping track of the individual participant’s contributions for transcription later on. Audio recording was also done. Each discussion lasted for 30-45minutes. The last 5-10minutes consisted of summarizing and recapping the response from the group. The discussions ended up with debriefing sessions to discuss the emerging themes and to assess if the information gathered met the objectives of the study.

### Data management and analysis

Each transcript was read and re read multiple times and the audio recordings of the FGDs was repeatedly listened to become familiar with the data in order to develop deep understanding of the data. The transcript was manually analyzed for data analysis using Thematic Content Analysis (TCA) by the researcher.

The textual data was desegregated into segments to examine the similarities and the differences in the data while grouping similar data. Similar data was coded which involved assigning a topic to the paragraph relating to the issue of interest in the study [26]. A list of code was developed with brief description of each code. New codes were assigned to passage that could not be coded with previous codes. Connections across codes were searched for to develop themes. New themes were created as the list of codes emerged. Whereas codes identify interesting information in your data, themes are broader and involve active interpretation of the codes and the data [27]. After reflection, their commonality was identified and a broad descriptor was assigned to them and some themes became sub themes of others. Finally, a master table was constructed containing themes, sub themes and quotes from the transcript. The hard and electronic copies of the data was securely stored.

### Measures to enhance trustworthiness

Scientific rigor was maintained in the qualitative study so that the views of the participants are accurately portrayed. The researcher adhered to criteria of ensuring trustworthiness to ensure rigor as described by Polit and Beck, (2012): credibility; dependability; conformability and; transferability [28]. Credibility was ensured through member checking where participant’s response was taken back to them for their viewpoint or alternative interpretation was done throughout data collection and through prolonged engagement with the participants’ Debriefing session was done with supervisor and peer scrutiny was undertaken. Participants were encouraged to be frank, rapport was developed and they were reminded that there are no right or wrong answers. The researcher kept on emphasizing her independent status so they could talk of their experiences without the fear of losing credibility in the eyes of a medical officer. To ensure dependability, all interviews had verbatim of transcription and direct quotation of the participation was used for presentation of the results and inclusion and exclusion criteria of the participants. Transferability was warranted through the use of sufficient contextual information about the fieldwork site was provided to enable reader to make the transfer. Tape recording and field notes ensured conformability.

### Ethical considerations

The ethical clearance to conduct the study was obtained from the College Health Research Ethic Committee (CHREC) at Fiji National University (FNU) and Fiji National Research Ethic Review Committee (FNRERC) and approval from the Sub-Divisional Medical Officer (SDMO), Ba was granted before the commencement of this study. The information sheet had all the relevant information regarding the study. Participants had a choice to participate and they were not under any obligation to participate and informed written consent was obtained from them. No personal details such as names or age of the participants was used to maintain anonymity and confidentiality. The researcher asked questions relevant to antenatal booking. The recorded interview was stored under lock and key in a safe cupboard.

## Results

### Characteristics of the HCWs

There was a total of 18 HCWs for the FGDs. According to the study findings, majority of the ANC service providers were female (88.9%), i-taukei (50%) and in the age group 25–35 years (66.7%). The mean age of the participants was 37.4±11.8years, with an age range of 26 to 64years. Majority were registered mid-wives and medical officers, comprising 77.8% of the FGDs and had been in the service for 2 to 10years (Table 1).

**Table 1:** Characteristic of the HCWs.

Variable	Frequency (%)
**Age** (mean ±SD)	37.4±11.8yrs
25-35yrs	12 (66.7)
36-45yrs	3 (16.7)
≥ 46yrs	3 (16.7)
**Ethnicity**	
i-taukei	9 (50)
Fijian of Indian descent	7 (38.9)
Others	2 (11.1)
**Gender**	
Female	16 (88.9)
Male	2 (11.1)
**Cadre**	
Medical officer	7 (38.9)
Registered Mid-wife	7 (38.9)
Registered Nurse	4 (22.2)
**Time in service (yrs)**	
2–10	13 (72.2)
> 10yrs	5 (27.8)

### Thematic analysis

After doing thematic analysis of the FGDs, 3 major themes were identified and these were knowledge of HCWs on starting ANC early, perceived barriers and enabling factors to timely initiation of ANC (Table 2). In this section, the cadre of the HCW will be described with MO, RN and MW (Medical officer, registered nurse and mid-wife respectively).

**Table 2:** Themes and sub themes of the FGDs with the HCWs.

Themes	Sub-themes	Categories
1. Knowledge of HCWs on starting ANC booking early	i) initiation of early ANC booking	Ideal timing
		Decision making
		Overall perception
	ii) Importance of early ANC booking	Confirm pregnancy
		calculate EDD
		Screening, preventive and curative
		advise and counselling
	iii) Late initiation of ANC booking	Timing of late ANC booking
		Consequences of late ANC booking
		Factors in general contributing
2. Perceived barriers to early initiation of ANC booking	i) Socio-demographic factors	Financial constraints
		lack of transportation
		employment status of the mother
		Ethnicity of the mother
		lack of partner and family support
		education level of the mother
	ii) Socio-cultural factors	Embarrassment
		Fear
		Inadequate knowledge
		Traditional beliefs and attitude of mother
	iii) Obstetric factors	Parity
		Undiagnosed pregnancy
		Past obstetric history
	iv) Health care factors	Accessibility
		Quality of service
		Human resource (attitude, knowledge and behavior of HCWs)
		Opening hours of clinic and waiting time
		Infrastructure
3. Enabling factors for early ANC booking	i) Health care factors	Improved quality of service
		Increased awareness of importance of early ANC
		Flexible opening hours of ANC
		Outreach visits
		First contact booking
		Multi-disciplinary approach
	ii) Client related factors	Increased family support;
		Feeling unwell in the current pregnancy
		Positive past obstetric history
		Fear of complication during delivery
	iii) community mobilization	Training of community health workers
		Involving church leaders and village headman’s
	iv) Policy considerations	Strengthen existing policies
		Provision of incentives
		Involvement of other stakeholders
		Transportation support

#### Theme 1—Knowledge of HCWS starting ANC booking early

The sub-themes deduced from the first theme were early ANC were initiation of early ANC, importance of early ANC and late initiation of ANC.

*Initiation of early ANC booking*. All the ANC service providers perceived ANC timing to be within the first trimester of pregnancy.

*“*…*the appropriate time for antenatal booking is from 8weeks to 12weeks as been required by all pregnant women*…*”*

(43yr old MW)

Majority of the participants expressed how the timing of ANC booking was not the decision of the pregnant mother only but also the spouse and close family members.

*“*…*decision making on timing of ANC is usually done by husbands and even in-laws*. *Few women are vocal and can plan and make decisions on their own*. *For indo-Fijian woman*, *family play a big role*, *on booking days a common sight is husband*, *mother in law accompanying the woman*…*”*

(55yr old MW)

A few of the participants highlighted how the health care factors are involved in the decision making of timing of initiation of ANC care.

*“*…*The SOPD sister in charge determines the time according to the average number of patients seen as to allow time for scan*, *blood test and bookwork*. *The SDMO and the recorders are also involved in the decision-making process*…*”*

(28yr old MO)

Overall, there was a positive perception of the ANC providers of starting ANC early.

*“*…*it is best to make early appointment as the doctor or mid-wife will take a detailed medical and family history as part of assessing your overall health*…*”*

(26yr old RN)

*Importance of early ANC booking*. Regarding respondent’s knowledge, they had good knowledge about importance of early ANC. There was a general understanding and recognition of importance of early ANC visits.

*“*…*early booking would mean that women’s pregnancy is officially confirmed through the scan*, *if any problem with the baby then it can be easily picked up*…*”*

(28yr old RN)

*“*…*it is good and appropriate time for all pregnant woman because there is ample time to follow up with them antenatal*. *For example*, *health education in regards to their diet*, *exercise*, *identification of pre-existing health*, *social problem*, *review of past obstetric problems*, *offering of screening tests*, *in case if any complications arise we can treat the minor problems and refer the major or complicated ones appropriately*…*”*

(43yr old MW)

Another medical officer had expressed the following:

*“*…*early booking also gives an estimation of gestational age*…*it is at the booking clinic that risk factors for abnormal pregnancy can be picked up*. *Abnormalities in blood pressure or sugars which may not have been picked up earlier can be detected here such as overt diabetes and hypertension*. *Cardiac anomalies*, *breast or cervical anomalies*, *STDS*, *Rh negative mothers can be seen at this time*…*”*

(32yr old MO)

*Initiation of late ANC booking*. The definition and understanding of late initiation of ANC care varied among the HCWS. Majority of the ANC service providers stated late booking was initiation of ANC in second trimester or third trimester (8 participants), 3 said initiation of care after 20 weeks, two said that it was either in third trimester or unbooked, two stated that it was after 12weeks and 1 said it was initiation of care after 16 weeks. 2 ANC providers did not define the timing of late.

The respondents understood the consequences of late ANC booking. It was highlighted that late booking will cause delayed diagnosis or missed diagnosis of certain disease and as a result the mothers will not be treated and the baby will suffer consequently.

*“*…*late booking is done in second and third trimester*. *It gets late to diagnose cases such as GDM (gestational diabetes) and serology results such as for syphilis*. *Therefore*, *mothers will be untreated putting the baby at higher risks*…*”*

(27yr old RN)

The ANC providers emphasized on the point that health education and counselling opportunities will be missed if a woman book late. Education and counselling are important for a pregnant mother to help the with the birth preparedness plan. Eventually with a lack of birth preparedness plan, mothers will end up with Birth Before Arrival (BBA).

*“*…*late booking means no opportunity to benefit from the screening tests*, *antenatal education and health advise and supported decision making regarding the place and choice of delivery*, *also increases infant and maternal mortality and morbidity*…*”*

(26yr old RN)

Another consequence of late booking that was cited by the ANC service providers was that it will delay in timely referral of high-risk pregnant mothers to specialist care.

*“*…*it also has been seen that late bookers are not evaluated properly in regards to social issues*, *risk factors and this delays early trans out of patients if needed*…*”*

(28yr old MO)

The participants expressed how there were a range of factors in general that could affect first trimester booking.

*“*…*family determines timing of booking for instance a pregnant mother has to see if baby sitter is available for mum to be able to leave older kids and come for booking*, *and then financial support for travelling*…*they also have to consider the issues in hospital such as too full and prolong waiting time sometimes discourage mothers from early booking*…*”*

(34yr old MO)

#### Theme 2—Perceived barriers to early initiation of ANC booking

Some of the barriers to early ANC booking identified by the HCWs were socio demographic, socio-cultural, obstetric and health care service related factors.

*Socio-demographic factors*. Even though the services at the hospital was free, the ANC service providers stated that women continued book late due to economic constraints such as lack of money to pay for the transportation to access health services.

*“*…*some mothers are more likely to present late for booking because they are not financially stable*, *they do not have the fare to come to hospital on time*…*”*

(34yr old MO)

*“*…*mothers book late because of financial constraint to pay for transportation*…*”*

(32yr old MO)

Participants also highlighted that being an employed mother could be a barrier to early antenatal booking and because of work commitment women may struggle to come for early ANC.

*“*…*Working mothers may not find time to come for booking*…*this can be because of their busy schedule or no one at work willing to cover for their shift*…*”*

(32yr old MO).

It was cited by the participants that even if women were not employed, they were too busy with the household chores and looking after other kids that led them to procrastinate their ANC booking.

*“*…*mothers have housework to do and they are too busy caring for other children*…*”*

(34yr old MO)

Some of the ANC providers also opined that ethnicity can be a factor for initiation of ANC.

*“*…*mostly Fijian of Indian descent would book at first trimester but on the other hand a Fiji Indian multip will book in third trimester*. *I-taukei women*, *only a few will book in first trimester most of them come in second/third trimester or are un booked*…*”*

(55yr old MW)

Moreover, the ANC service providers revealed that lack of partner and family support was one of the factors that can contribute to delay in ANC booking among mothers.

*“*…*mothers might delay their booking because they want their spouse to accompany them*, *especially the first-time mother as it is a totally new experience for them*, *therefore they continue to wait until their spouse has time to accompany them*…*some end up coming with no spouse even though they delay booking for the partner*…*”*

(32yr old IT MW)

Education level of the pregnant women also determines the timing of booking as was opined by the participants:

*“*…*some educated women would book as soon as they miss a menses while some will not think it’s necessary*…*”*

(55yr old MW)

*Socio-cultural factors*. Participants noted embarrassment in women who got pregnant at an early age or at older age as a barrier to early ANC booking. Upon probing on reasons for embarrassment it was pointed out that the nurses discouraged them as they were at higher risk of obstetric complications.

*“*…*the youngest patient I have booked is 15yrs old*. *I have heard from some mums that they are sometimes shy or feel abnormal about booking because they are either over 40yrs or have more than 5kids*…*”*

(34yr old MO)

Moreover, the ANC service providers also highlighted that fear is a contributing factor to late ANC booking.

*“*…*fear of the unknown*, *fear of the health care providers*, *fear of previous experience is also a factor*.…*”*

(55yr old MW)

Lack of knowledge on the importance of early ANC booking of the mothers was stated by participants as hindrance to early ANC booking. Health care providers reported that women do not know the optimal time to start ANC and may attend only to secure booking for obstetric service.

*“*…*the opinion and knowledge about the value of early ANC of our pregnant mothers might be really low*…*they might not even know why they have to come early for booking*…*”*

(27yr old RN)

Health care providers cited that general lack of knowledge about the early signs of pregnancy also contributed to late booking.

*“*…*Mothers come late simply because they are unaware that they are pregnant*…*they fail to recognize the signs of pregnancy; they continue to have their menses or they conceive while on family planning*. *Family planning such as Depo injection gives amenorrhea so even if they do conceive the amenorrhea is attributed to Depo*…*”*

(28yr old MO)

Beliefs and attitude of pregnant mothers was also highlighted as a contributing factor to late booking. The participants expressed how the attitude of the pregnant mothers influences the timing of ANC booking. This is what a mid-wife had to say:

*“*…*having a ‘yes I must attend ANC or no I don’t care’ attitude influences the timing*. *Multiparous know a lot*!!…*some multips because they have been through the process once or several times think there is no need to attend early booking as long as they attend booking anytime during the pregnancy*…*”*

(40yr old MW)

This might mean if nothing has changed the perceived negative attitude of the HCW and the poor quality of service by the mother will be a barrier to early booking.

The ANC providers revealed that many women access ANC late because of ignorance and that they want to make fewer ANC visit.

*“*…*for some mums its ok to come for early booking at that time*. *But for most of the mums who have more than 2 kids they prefer coming in when they are in 2*^*nd*^ *or 3*^*rd*^ *trimester because they can attend less number of clinics*…*”*

(32yr old MO)

It was opined by a participant how community norms can be a barrier to early initiation of ANC as few women were still engulfed in the traditional past. She commented that:

*“*…*for an Indian female who conceives after several years of her marriage*, *she might be advised by the elders of her family or community not to reveal her pregnancy to anyone at least for 3 months as it might bring bad luck and they can end up having a miscarriage*…*so going for scan and early booking will mean revealing the pregnancy to others hence they will wait for at least 3 months*…*”*

(28yr old MO)

*Obstetric factors*. Multi parity was also stated as a barrier to early ANC use by the participants. Participants expressed that multiparous mothers, especially the older ones purposefully postpone ANC and did not value ANC services in the early. This was attributed to women’s knowledge gained in previous pregnancies and their prior experience.

*“*…*a Fiji Indian multiparous would book in third trimester*…*some mulitps (mothers who have given 2 or more previous births) because they have been through the process once or several times think there is no need to attend early booking as long as they attend booking anytime during the pregnancy*…*multips know the booking process are very long and are reluctant to come early*…*”*

(55yr old MW)

Undiagnosed pregnancy was also seen by the participants as a hindrance to early booking.

*“*…*because they don’t even know that they are pregnant*…”

(27yr old RN)

It was highlighted in the FGDs that a woman’s past obstetric history was also a determinant of initiation of ANC care

*“*…*a previous experience with an HCW might create fear*, *previous experience such as still birth or miscarriage or difficult delivery may make one scared or unwilling to come early for booking*…*”*

(40yr old MW)

*Health care service factors*. Another factor cited by the participants in accessing timely ANC care was long distance to health facility. This is worsened by lack of transport due to the poor road conditions.

*“*…*some give geographical location as a reason for booking late; the health facility maybe far from patient’s home*, *transportation maybe difficult*…*”*

(30yr old RN)

Unfavorable service at the health care facility was highlighted as a contributing factor in delaying first ANC visit. One participant said:

*“*…*not being able to develop a good rapport with our patients can contribute to late booking*…*many of ourselves are unapproachable to these anxious mothers*…*”*

(34yr old MO)

All cadre of the ANC providers cited that women might be delaying ANC booking because of the negative attitude of the HCWs. The following were some of their responses:

*“*…*negative attitudes of the health care providers*. *A bad history of ANC can have an effect on patient especially when they are pregnant and they have to attend clinics again*. *This bad experience can delay them in coming for early booking*…*”*

(28yr old MO)

In addition to that, it was pointed out that both miscommunications such as contradictory information about the time of booking and lack of communication to pregnant mothers about booking can be influencing factors to timely initiation of ANC service.

*“*…*misinformation from the non-medical hospital staff*. *Some women were turned away and were told to book once they are more than 20weeks which defeats the purpose of our advice of early booking*…*”*

(28yr old MO)

It was further highlighted by the participants that lack of awareness and advocacy on early booking by the health system was one of the many factors contributing to delaying ANC.

*“*…*I think our health system lacks the advocating of early booking for mothers*…*”*

(28yr old MO)

Lack of pregnancy test kit in the hospital contributes to late ANC booking as it prolongs the period of undiagnosed pregnancy for a few mothers.

*“*…*mothers delay their booking because of late diagnosis of pregnancy due lack of supplies of pregnancy test kit in hospitals*…*”*

(27yr old RN)

Participants also highlighted that opening hours of ANC booking was very limited because of which patients were not booking on time as indicated by the following quotation:

*“*…*the timing of our booking clinics*. *Some women are unable to make time to attend in the morning due to transportation and work commitment*…*”*

(28yr old MO)

Some opined that pregnant women had to wait to very long and sometimes have to be turned away because of the long procedure of booking and limited staffs to see the patients.

*“*…*allocation time—from 8am to 1pm is too short*. *Often mothers are turned away by 11;30 or 12 mid-day due to the process they have to go through*. *Then the waiting time in the lab*, *they have to pick up numbers and this often delays them to be examined by the doctors*…*”*

(63yr old MW)

The ANC booking room is also used for other clinics such as family planning, pap smear and NCD clinic. The participants expressed this can be a contributing factor to delayed ANC booking.

*“*…*We have had cases when on booking days the woman comes after the allocated time and because we are doing pap smear*, *Jadelle procedures we had to send them back*…*”*

(55yr old MW)

Furthermore, a few stated that the ANC booking room was too congested which led to lack of privacy that can be a contributing factor for delaying ANC booking.

*“*…*the space is too small to provide the necessary examination to pregnant women*, *can only accommodate 2 nurses*, *the dietician has to squeeze in*…*”*

(63yr old MW)

#### Theme 3—Enabling factors for early ANC booking

The sub-themes for factors that can enable women to initiate ANC early were health related factors, client related factors, community mobilization and policy consideration.

*Health care service factors*. The ANC service providers stated that due to their workload, sometimes their behavior towards the patients changes and they also understood that their behavior and attitude towards the pregnant women influences a woman’s decision to book on time. They cited that if their approach towards the client was good, then more mothers might turn for early booking. The following were some of their ideas that will enable timely booking:

*“*…*ä competent and organized health team who assists the woman when she comes to hospital*…*shaving a good system in place there should be efficiency in the management of patients*, *availability of resources*, *a system that allows woman to have access to health care whenever she needs it*…*”*

(55yr old MW)

It was highlighted by the participants that the ANC booking area needs to be bigger to accommodate more people to decrease the waiting time for the patients as the work flow will be easier. Having more staffs during booking was also considered an enabling factor to early initiation of ANC care

“I think the area that needs to be addressed is if a bigger room was given for ANC and if the lab technicians can allocate some-one specifically to take bloods from the ANC mums for faster results of the blood tests to accommodate workflow for ANC mums and shorten the waiting time for mums”

(39yr old MW)

It was also brought up in the discussion as how having an ANC clinic on its own will enable more mothers to be booked. The mid-wife expressed:

*“*…*having the antenatal clinic on its own unlike our setting in Ba will enable us to do bookings apart from booking days when the women come to hospital*…”

(55yr old MW)

The participants highlighted that the health center, nursing station and the zone nurses play an important role in enabling mothers to be booked on time.

*“*…*the public health team should identify the un booked mothers targeting those who are more likely to miss ANC such as the adolescents*, *single and poor mothers*…*”*

(28yr old MO)

The ANC providers cited that a flexible opening of the clinic hours will enable mothers to initiate timely ANC.

*“*…*reassess the timing of booking clinic as to make it more convenient to the mother*, *ANC booking is timed from 8am to 11am*. *I personally think there should be another session in the afternoon*. *There are mothers who will find it difficult to attend clinic during the allocated time due to work commitment and transportation issues*…*”*

(28yr old MO)

It was highlighted that the opportunities for early booking can be maximized by taking advantages of the existing programs such as outreach activities targeting women on child bearing age. The respondents of the FGDs felt that one of the enabling factors for early initiation of ANC care can be through provision of ANC service to the mothers who have difficulties in accessing the health care service. This for instance includes booking pregnant mothers during home visits and outreach clinics.

*“*…*we as health personnel can help mothers with early booking by making arrangements to go to them if they are unable to come to hospital (zone nurse)*

(33yr old RN)

Moreover, it was highlighted that first contact booking should be done. Any pregnant mother presenting to any health facility should be registered and booked from there and then referred to BMH for further investigations. This way the mother will be committed to booking and will be less likely to delay ANC booking.

*“*…*woman who attend their health facility*, *irrespective of being a health centre or nursing station should be booked there and then referred to the base hospital for further clinics*…*”*

(55yr old MW)

The ANC service providers discussed the benefits of multi-disciplinary approach to ANC as efficient and effective way to encourage woman to attend. They discussed their working relationship with social workers, mental health nurses, counsellors, dieticians, public health nurses and private physicians.

*“*…*women have to face so many barriers before they reach our doorsteps*, *so I think once they are here we should do a good job meeting their needs*, *such as referring them to the dietician*, *to mental health nurse if she is too stressed*…*and with first visit you can still follow them up through the zone nurses who know that patient better*. *In that way they will feel cared for and will discuss their experience with other women who might then be motivated to come for ANC*…*”*

*(34yr old MO)*

*Client related factors*. Feeling unwell during the current pregnancy was cited as an influencing factor for early ANC booking. Participants explained that pregnant women who were unwell would want to seek treatment from the hospital and thus come early for ANC booking.

*“*…*women who are unsure or start having vomiting as a complaint first come to GOPD (general outpatient department) then they are sent for scan which confirms pregnancy then booking is done after the MO (medical officer) advises them*…*”*

(27yr old RN)

Participants also said that fear of complications during delivery can be a motivating factor to early ANC booking. Participants expressed how a woman will want to book early for expert assistance if she had complications during her previous pregnancies.

*“*…*a mother might be having her 2*^*nd*^ *or even 5*^*th*^ *or 6*^*th*^ *child and had bad experience with their last or previous delivery will come for early booking*…*”*

(34yr old MO)

The participants highlighted that being a primiparous mother can be an enabling factor for early ANC care.

*“*…*some first-time mums are enthusiastic about their pregnancy*. *The joy of becoming first time mothers*, *they book early so they learn about the development of the baby*…*”*

(34yr old MO)

Furthermore, it was gathered from the discussions that having a good family and partner support might enable mothers to book early. The participants understood that it wasn’t the pregnant woman’s sole decision to come for timely booking and that their decision was influenced by their spouse and family members. Family members include in-laws, siblings who have previously accessed the health care from the ANC clinic.

*“*…*outreaches can do heath talks on importance of early booking so people can understand and support their wives/mothers not only financially but also socially and emotionally enabling them to come for early booking*…*”*

(34yr old MO)

*Community mobilization*. Participants highlighted that it was important to train the community health workers to identify the pregnant women in the community and counsel on the need for early ANC booking and skilled care at birth. CHW will create a link between the community and the health care system while reinforcing the health messages.

*“*…*utilize the community health workers*, *they know their communities well and should send these mums to hospital early*…*”*

(40yr old MW)

Another participant had this to say:

*“*…*training of CHW on Birth Preparedness Plan and Complication plan was conducted and this needs to be re-visited*…*”*

(63yr old MW)

Moreover, improved communication among the ANC service providers, CHW and the traditional birth assistants (TBAs) may improve early initiation of ANC care. The participants cited that having the knowledge of the service being offered and the importance of early ANC booking will strongly influence a pregnant woman’s decision to seek timely ANC. The ANC service providers recommended that increased awareness on the importance of early ANC booking through the TBAs and CHWs will enable mothers to book on time.

*“*…*provide community-based information*, *education and communication on ANC and its right time of commencement*…. *raise awareness to the public to educate them on the importance of early booking*…*have pamphlets in ANC waiting area have advertisement on TV on early booking*…*”*

(63yr old MW)

Social support provided by the members of the public also entails community resource. These includes support at workplace and support by school teachers through Ministry of Education.

*“*…*teachers and lectures should be more supportive of their pregnant student*, *they should be released from their classes to let the students come for booking*, *also I think the reproductive curriculum in secondary schools should focus on missed menses and what to be done in case if missed menses*….*”*

(34yr old MO)

*Policy consideration*. The participants expressed the existing guidelines should be updated with training of the ANC service providers.

*“*…*our current clinical practice guidelines states that booking more than 24 weeks is considered late booking*…*they have to relook at that so that we the HCWs have the right information to give the right advice to the pregnant women*…*”*

(32yr old MO)

Some participants cited how promotions and incentives by the government can enable mothers to book early.

*“*…*health ministry should have a legislation on which AOG (age of gestation) pregnant mothers should come for booking because at the moment even incentives are given but still late booking is happening*. *Incentives should be provided to all pregnant women instead of rural mums only*. *Current incentive for social welfare assistance is not given to civil servants and town area residents*.

(39yr old MW)

The FGDs also showed that social welfare support can also assist mothers to book on time.

*“*…*identify those mothers who has difficulties in making ANC appointments and helping them find their way to hospital*…*they can even assist is buying necessities for the baby and the mother*…*”*

(34yr old MO)

It was also highlighted that mothers could be assisted with transportation by offering bus passes, bus tickets or running of vans to pick them up.

*“*…*the current social welfare that a few families get is not enough for mothers to accommodate transportation*. *Transportation can be a big issue*. *Government can help like give bus pass or free tickets or transport can be arranged for them to be pick up from home such as carrier vans*…*”*

(39yr old RN)

## Discussion

The findings of the study revealed that the HCWs in Ba had adequate knowledge on the timing and importance of early initiation of ANC. However, they cited that the decision of first ANC visit is complex as it is influenced by many other socio-demographics, cultural, obstetric and health care related factors. The definition of late booking for HCWs ranged from first to third trimester and they seemed to understand the consequences of late booking. However, they failed to disseminate this knowledge to the pregnant women most of the time. There is a need for in-service training on ANC within the hospital setting to increase knowledge and to create more awareness on ANC care and importance of early booking. The finding of Mother Safe Hospital Initiatives (MSHI) audits which is conducted monthly should be disseminated to staffs at the grass root level.

Some of the socio demographic barriers reported in the current study were financial constraints, lack of transportation, employment status, education level and ethnicity of the mother and lack of family and partner support. These findings confirm results from previous studies done in sub-Saharan African countries [29].

The current study revealed that financial problem was one of the barriers to initiating timely ANC. These financial constraints are related to barriers such as transportation cost and the cost of obtaining care. Other studies also highlighted that women who are relatively wealthy are less likely to be perturbed by the total cost of early initiation of pregnancy [29]. The implication of this is that ANC booking should be done through outreach visits by the zone nurses, nursing station should be equipped with basic resources for antenatal booking and women should be booked any time she presents to a health care facility.

HCWs opined that education level of the pregnant women also affects their decision on timely booking. They revealed that because educated mothers understand the complications of late booking, they would present early for booking. In accordance to that, other studies done in Northern Ethiopia and in Bhutan also showed that women with primary education level were 2.2 times more likely to initiate ANC late than those with college, diploma and higher level of education [30, 31]. Educated women might be booking early as they are well informed about the benefits of initiating ANC early whereas illiterate women might have little understanding of the importance of early ANC. Therefore, awareness on early ANC booking should be created using Information, Education and Communication (IEC) materials for the uneducated women in the reproductive age group. Moreover, there is need to strengthen and encourage women’s education to higher levels as education level plays an important social role [29].

HCWs enlightened that employment status does influence timing of booking. They revealed that being employed can have either positive or negative influence on the timing of ANC booking. A study in Italy showed that unemployed women were booking later than employed women [32]. Unemployed women might be housewives and they have a lot of workload and hence lack of time to go for booking. It could also be because they have lower education level or that they are non-permanent employee and ANC booking means spending a lot of time in clinic, away from work. Contrary to that, few employed women were booking late according to the HCWs. One of the explanations for this could be that these women are busy with economic activities to cater for their families or to earn enough money for transportation to health facility for ANC clinics or to buy new clothes for the newborn. Thus, understanding the daily lives of the women in the community is important to inform interventions to ensure early initiation of ANC. Outreach and shift clinics should also target pregnant women. Health facilities should book mothers after the normal working hours, at a time that is convenient to the pregnant women.

It was established from the study that I-taukei women did not start ANC on time as they wait for their church pastors or TBA or mother-in-law to come and give them advice. This leads to pregnancy being secret until it reaches 3 or 4 months old. The HCWs also opined how Fijian of Indian descent mothers who conceived after several years of marriage wish to keep pregnancy secret until 3 months. This was also noted in studies conducted in Malawai, Republic of Congo and Zambia show that culture and religion influence ANC attendance and the practices attached to these beliefs delay initiation of ANC [33, 34]. The social beliefs or the myths associated with pregnancy should be addressed through the use of village health workers, *turaga ni koros*, church leaders and pastors. The *Bose Ni Tikina* meetings should be used as platforms to address the complications of late booking with evidence given from local setting. A systematic review of determinants of ANC utilization in sub-Saharan Africa showed within country ethnic differences was shown to influence attending at least one ANC [35]. Various cultural factors might be related to ethnicity which acts as barriers to initiate timely ANC care. Culture plays an integral part in lots of decision making in PICs as preferences and beliefs are governed by the ethnic roots of Pacific Islanders [36].

Within ethnicity comes language barrier which can be contributing towards late ANC booking [37]. Studies involving broad mix of ethnic population have shown language barrier is a prior barrier to booking. Difficulty in communicating effectively with the health professionals might leave women unable to understand and access the service available to them. Majority of the communication in the current study setting is in English language which might not be understood by the I-taukei mothers. The design of public health interventions to increase the timeliness of ANC initiation will therefore require a greater understanding of pregnant women’s differences in perceptions across ethnic groups. During awareness campaign it is important to take nurses or doctors who are fluent in the language that the community is speaking. If mass media is used to create awareness, then it should be in different languages.

Furthermore, the HCWS revealed that lack of partner or family support also contributed to delay in seeking ANC as they observed the partners hardly accompanied the pregnant women for booking clinics. They highlighted that some women delay ANC booking because their husbands refuse to accompany them. Lack of partner support contributing to late ANC booking was also reported by other studies [38, 39]. Partner involvement in terms of accompanying the women for ANC or even initiating or supporting early use of ANC may have an important impact on early ANC booking. Fiji should have a policy in place that promotes male involvement in ANC so that women are accompanied by male partner. ANC clinic should be made “male friendly “to increase male participation and policies should be relooked at to increase male involvement in maternal and child health care. Targeting mother-in-law and husbands in health promotion and educational intervention could be a promising strategy to improve early initiation of ANC services in Ba.

Embarrassment, fear or trust was reported as barriers to health seeking behavior of mothers in various studies [40–42]. Likewise, the current research showed pregnant mothers were embarrassed to come for ANC booking if they were either too young or too old. Fear of the health care provider and fear of previous experience by the pregnant woman was also reported by the HCWs as barriers to early initiation of ANC. ANC attendance is responsive to the attitude of the health staff; positive or negative impact on the ANC turnout. Current study revealed that it was the negative attitude of the HCWs that prevented the mothers to book on time. Poor relationships with health care providers or negative experiences at the clinic deterred women from attending clinic. Therefore, during subsequent ANC visits, the HCWs should be talking to women to build rapport while advising them the importance of early booking in upcoming pregnancies. HCWs should not be judgmental and should be treating patients with respect while maintaining confidentiality. ANC clinics should have patient surveys or feedback about the health services offered as means of improving the quality of care delivered. Improving the perceived quality of care given to pregnant mothers can improve early ANC booking. HCWs need in service training on customer care and there should be zero tolerance for disrespectful behavior. Interventions policy should be developed to consistently address disrespectful behavior. Levels of interventions might start with coaching and proceed to progressive discipline as warranted. The intervention policy should clearly articulate the behaviors or repeated behaviors that will be referred for disciplinary action, and how and when the disciplinary process will start.

Lack of privacy in the ANC room was reported as one of the barriers to early booking by the ANC service providers. This lack of privacy might demotivate women from booking early. The HCWs further highlighted that unfavorable service offered to the patients affected their timing of ANC booking. The ANC room used in the study setting was small that was shared amongst the mid-wife, doctor, nurse and occasionally a family planning nurse. Management should look into relocating ANC to another location with enough space that ensures patient privacy. However, if location cannot be changed then every effort should be made to maintain privacy and see one patient at a time.

HCWs also expressed the attitude of the mothers influenced on the timing of their booking. In the current study HCWS stated women were starting ANC late so they have to attend less ANC visits before delivery. The HCWs revealed that many women access ANC late because of ignorance and that they want to make fewer ANC visits. The results corroborate with those findings in Papua New Guinea where most women reported to have started ANC late with an aim of reducing number of visits before delivery [43]. There is a general perception among women that once you start attending clinic, you will be compelled to attend follow-up. Women should be advised on the need for the follow up visits, they need to understand what difference will the follow up visits make to their pregnancy and this should be done on case by case basis. More time should be spent with pregnant women for them to understand their own pregnancy instead of rushing through clinics to clear the crowd.

In addition to that, experience of previous pregnancy also had an impact on timing of booking in the current study. Women who had positive outcome did not perceive any benefit from early booking and they postponed their ANC booking. Therefore, for postpartum mothers with no complications, it is important to counsel them on the importance of early booking upon discharge. They have to understand that by coming early for booking the chances of positive outcome in next pregnancy would be higher. The information on early ANC booking should start from the postnatal clinics. Our current practice is following up of complicated cases closely postpartum.

HCWs also highlighted that multiparous women were delaying initiation of ANC. Similarly, the analysis of Rwands DHS data further revealed that having more than 4 children was significantly associated with delayed ANC [44]. Multiparous women might also be finding it difficult to look after other children as was highlighted in the current study. Thus, our ANC setting should be such that it is able to cater for preschoolers. It should have kid’s fun club or at least a baby sitter to look after the young kids while women get her booking done. Furthermore, this also implies the importance of having accessible family planning services to help couples achieve their desired number of children and for child spacing. For primigravida women in our study, community-based source of information on when to start ANC is needed to encourage early ANC booking and this should be emphasized in subsequent pregnancies.

Undiagnosed or late diagnosis of pregnancy was also affecting early booking as opined by the HCWs. Education and awareness of early signs of pregnancy can improve diagnosis of pregnancy and hence earlier booking. This finding also implies that pregnancy test kit should be available in the health facility for earlier diagnosis of pregnancy especially for the poor who cannot afford to buy the kit and wait for scan to confirm pregnancy.

Long waiting time was also reported by HCWs as one of the factors that delays ANC booking. Other studies also showed that the long waiting time by often discouraged pregnant women from booking on time [9, 45–47]. Moreover, the HCWS opined that the fixed opening hours of the ANC booking can also affect timing of ANC booking. According to the HCWs inconsistent message given to pregnant women in the current study also affected initiation of ANC care. This finding was consistent with the findings of other studies. Women who received correct information on accurate time of booking were more likely to book early for ANC [48]. Furthermore, lack of advice from the HCWs itself was a barrier to early initiation of ANC in the present study. HCWs therefore play a pivotal role in advocating for early booking. They have to give the right information to women with a very clear and concise message.

The HCWs recommended that the opening of the ANC clinic should be relooked at, ANC booking should be done by the medical officer who first confirms pregnancy. They suggested if awareness on early booking be raised not only in the hospital setting through the use of IEC materials but also in the community at a large through the use of mass media. Service should be delivered at home through outreach and home visits. It was also recommended to train the CHWs to identify the pregnant women in the community and counsel on the need for early ANC booking and skilled care at birth. Strengthening of existing policies to improve first trimester booking was suggested by the HCWs with a multi-disciplinary approach. A few policy considerations such as provision of transportation for women to be fetched for booking, incentives for early bookers might reduce the burden of late antenatal booking as was suggested by the HCWs [49].

### Limitations

Study limitation relate mainly to the sample in that the majority were females and that the view of male HCWs might have been under reported. There was an over representation of the mid-wives and the doctors than the registered nurses who usually are the frontiers of health service delivery. Since the study was conducted with the HCWs, there is a possibility of underreporting of health care related factors. The HCWs spoke based on their experience in the current setting, thus will have varying degree of transferability in other settings. Therefore, the study findings can only be generalized to the parent population and not across other setting where different geographical, cultural and health service delivery factors will affect early initiation of ANC. Some of the issues which could have added further value to the study were not explored in depth due to time constraint. This included issues such as danger signs of early pregnancy. The opinions of the FGDs are not of the individuals but of the wider group which might not give a true picture as members might not have expressed their true opinions. Two participants who initially agreed to participate did not turn up on the day of the FGDs therefore decrease the size of 2 groups. The findings of the FGDs are not generalizable. While more studies covering different geographical settings would be helpful for evidence based policy-making, this study provides an in-depth understanding of various factors which influence the timing of the first ANC attendance.

## Conclusion

Late antenatal booking is common in Ba and even though the HCWs have positive perception of early antenatal booking, they are prevented from doing it so as the timing of ANC visit is influenced by a spectrum of causes from socio-cultural, socio-demographic, obstetric and service delivery factors. The barriers to early ANC are both an opportunity and a challenge to strengthen and review the maternal services. The enabling factors should be reinforced from an individual level to the health system and the general context. The HCWs should be trained on customer care so that they can treat patients with respect and are approachable. The traditional leaders in conjunction with community health workers should come up with laws which can assist women to come for early ANC by addressing the socio-cultural practice. There is a need for increased awareness on timing and importance of early ANC booking. Women should be empowered to make their own decisions concerning their health and the health of their unborn child. Mobile clinics or outreach clinic should also do ANC booking for hard to reach women for easier accessibility and the opening hours of ANC should be extended.

## Supporting information

S1 File (DOCX)
