# Supplementary material for: Perception of Health Care Workers (HCWs) towards early antenatal booking in Fiji: A qualitative study
Source: PLoS One. 2022 Nov 28;17(11):e0276805. doi: 10.1371/journal.pone.0276805 (PMC9704654; doi:10.1371/journal.pone.0276805)
Supplement: S1 File — (DOCX) [file pone.0276805.s001.docx]

## Topic Guide for FGDs

FGD Group:

Gender

Name:

Occupation:

Age:

Years of experience:

1. Describe your experience antenatal booking at Ba Mission Hospital
2. When do you think is the appropriate time for antenatal booking?
3. What do you think about this timing for your clients? Who do you think is involved in the decision making?
4. What do you know about early and late booking?
5. What do you think are some health care related factors which delays early booking?

What are some areas of support required to enable early booking of pregnancy?
